# Capacity building for critical care skills training provision in resource limited settings: the nursing intensive care skills training (nicst) project

**DOI:** 10.1186/2197-425X-3-S1-A444

**Published:** 2015-10-01

**Authors:** T Stephens, A Beane, AP De Silva, J Welch, C Sigera, S De Alwis, P Athapattu, L Peiris, S Siriwardana, KSA Jayasinghe, A Dondorp, R Haniffa

**Affiliations:** Barts Health NHS Trust, Critical Care Research Team, London, United Kingdom; Queen Mary University of London, William Harvey Institute, LONDON, United Kingdom; Barts Health NHS Trust, Adult Critical Care Department, LONDON, United Kingdom; National Intensive Care Surveillance, Colombo, Sri Lanka; University College London Hospital, London, United Kingdom; Ministry of Health, Office of Deputy Director General (Education, Training and Research), Colombo, Sri Lanka; Nursing Council of Sri Lanka, Colombo, Sri Lanka; Department of Clinical Medicine, Faculty of Medicine, University of Colombo, Colombo, Sri Lanka; Mahidol Oxford Tropical Medicine Research Unit (MORU), Bangkok, Thailand

## Introduction

The availability of high quality critical care is increasingly recognised as a global health problem [1, 2]. The ability of any health system to scale-up delivery of effective critical care services will be limited by critical care training capacity.

## Objectives

Evaluation of a capacity building project to enable local nurses to deliver critical care training courses in a resource limited setting (Sri Lanka).

## Methods

A short critical care course for nurses (Nursing Intensive Care Skills Training; NICST) was co-designed & delivered by specialist overseas nurse trainers in partnership with Sri Lankan nurse tutors. The impact of the course was assessed using: a pre & post course Multiple Choice Questionnaire (MCQ); a post course Objective Clinical Skills Assessment (OSCA) station & post course feedback questionnaires. Training was delivered in 7 blocks, from June 2013 to November 2014. A graded handover of training responsibilities occurred with lectures, skills stations & workshops increasingly delivered by local rather than overseas faculty. From March 2014 onwards the course was delivered entirely by local faculty, with coaching by overseas faculty. This process was coordinated with a Train the Trainers (TTT) programme for local faculty. The impact of the TTT for building effective training capacity was assessed by analysing the NICST course participant MCQ marks over time.

## Results

**Figure 1:**
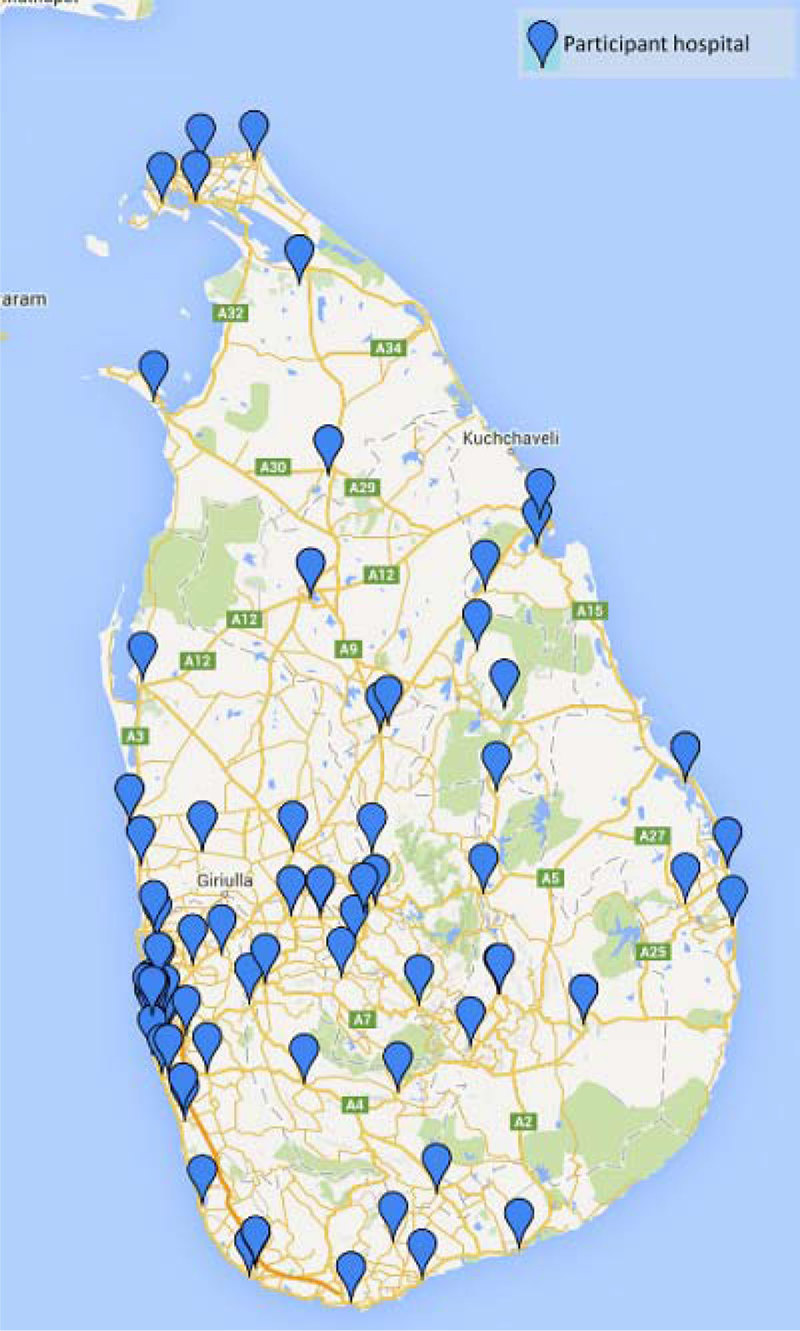
**NICST reach island wide.**

**Figure 2:**
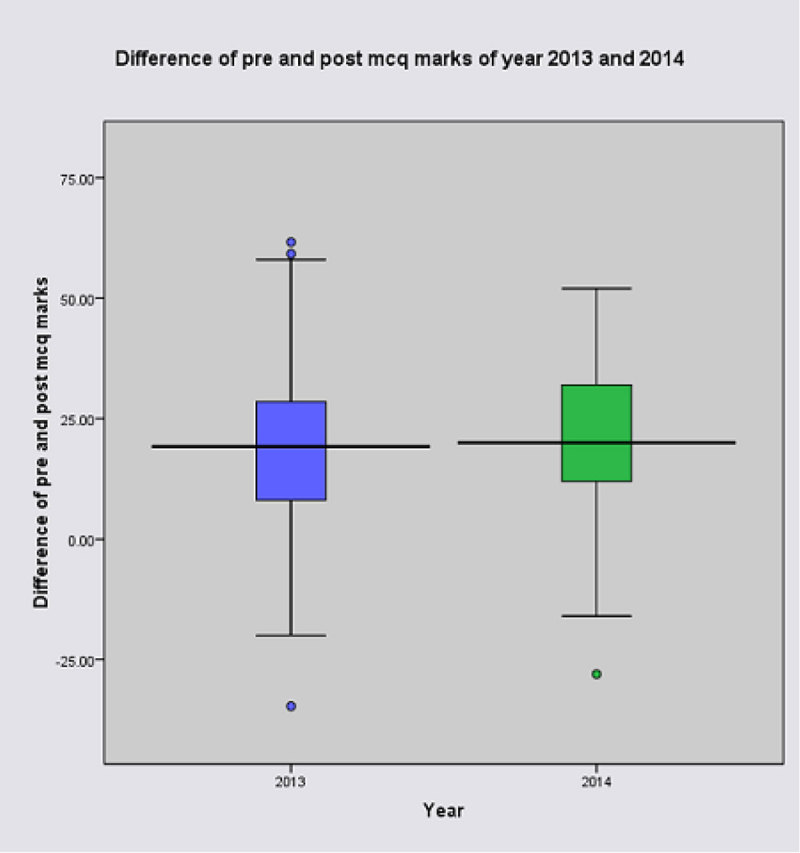
**Difference of MCQ marks: 2013/2014.**

In total 584 ICU nurses were trained over 16 NICST courses. Figure 1 shows equitable distribution of training provision between rural & urban areas across the island. Participant post MCQ scores were significantly higher when compared to pre MCQ (P < 0.0001; Wilcoxon sign rank test) across all courses. More than 64% passed the OSCA in each course. Participant feedback across all courses was positive with 98% agreeing that the course was relevant to their practice & 96% agreeing that the course was worthwhile. Comparison of MCQ results between 2013 (more overseas faculty input) & 2014 (NICST run by entirely by local faculty) showed no significant difference (p = 0.186; independent sample t test; Figure 2). This suggests that the local faculty and organisational development required to deliver NICST effectively has occurred.

## Conclusions

This training is highly rated by participants & is effective in improving clinical knowledge. The roll out of NICST has also fostered a new community of practice amongst the nurse tutor workforce in Sri Lanka, focused on delivering improved critical care skills training. The TTT approach provides sustainable training capacity within the local workforce. This approach may be of use in other resource limited settings.
